# Vancomycin-loaded HPMC microparticles embedded within injectable thermosensitive chitosan hydrogels

**DOI:** 10.1007/s40204-017-0066-x

**Published:** 2017-04-26

**Authors:** M. Mahmoudian, F. Ganji

**Affiliations:** Department of Biomedical Engineering, Faculty of Chemical Engineering, Tarbiat Modares University, P. O. Box 14115-114, Tehran, Islamic Republic of Iran

**Keywords:** Chitosan, HPMC, Hydrogel, Microparticles, Vancomycin hydrochloride

## Abstract

Antibiotic use is an essential method for the treatment of bacterial infections. In certain cases, antibiotics are not effective because of the distribution problems caused by physiological barriers in the body. Such problems are thought to be minimized by development of sustained release systems which involve implantation of antibiotic loaded polymeric systems directly to the site of infection. In this work, a new composite vancomycin hydrochloride release system based on HPMC microparticles and chitosan/glycerophosphate (Ch/Gp) thermosensitive hydrogel was designed for the aim of local treatment of osteomyelitis. Vancomycin-loaded HPMC microparticles (Van-HPMCs) were prepared by spray drying method. The SEM results showed that these particles had a mean diameter of 1.5–6.4 μm with a narrow size distribution and homogeneous particle production. Their drug encapsulation efficiency was 72.6%. The Van-HPMCs were embedded in an injectable Ch/Gp solution to introduce a composite drug release platform (Van/HPMC-Ch/Gp). In vitro release studies indicated that inclusion of the Van-HPMCs into the Ch/Gp hydrogel caused a reduction in both the release rate and total amount of vancomycin release, which suggests that HPMC microparticles entrapped into the Ch/Gp hydrogels showed more suitable sustained release kinetics for local antibiotics delivery.

## Introduction

Osteomyelitis is a bone disease caused by bacterial infection. The disease is characterized by a bacterial plaque formation around the infected area and is classified as either acute or chronic depending on the duration of the infection (Xiong et al. 2014). In treatment process, it is desirable to maintain a therapeutic level of antibiotic for at least 4–6 weeks to control the infection (Balmayor et al. 2012; Soundrapandian et al. 2009, 2010). Vancomycin is a glycopeptide antibiotic with a strong bactericidal activity against many Gram-positive bacterial infections (Ravelingien et al. 2010). Vancomycin hydrochloride with a molecular weight of 1486 is a brownish powder that is highly soluble in water. It is preferred for the bacterial infections in patients allergic to beta-lactam antibiotics and has been used clinically for over 50 years. Pharmacokinetically, vancomycin hydrochloride has a long elimination half-life of 5–11 h for patients with normal renal functions and demonstrates a post antibiotic effect lasting between 1 and 6 h. Owing to the localized plaque, it is very difficult to treat osteomyelitis via systemic vancomycin administration, because the bacterial plaque would disable diffusion of the antibiotic from the blood to the site of infection. The emerging drug delivery technologies alleviate such disadvantages of traditional therapy of osteomyelitis where antibiotic loaded polymeric formulations are implanted to the infected area (Darestani-Farahani et al. 2016; Xiong et al. 2014).

In situ forming thermosensitive hydrogels are an important technology for sustained drug delivery (Peng et al. 2013; Ganji and Vasheghani-Farahani 2009; Ngwuluka 2010). Thermosensitive hydrogel is a free-flowing liquid under room temperature and can form a semi-solid gel at body temperature and thus, acts as a barrier for the release of the embedded drugs. Thermosensitive hydrogels made of various polymers and biopolymers have been widely investigated as a system of local drug delivery. Among them, chitosan/glycerophosphate (Ch/Gp) hydrogel has emerged as a unique kind of injectable thermosensitive hydrogel owing to its excellent properties, including ready availability, biocompatibility, biodegradability, as well as non-toxicity (Ghasemi-Tahrir et al. 2015; Li et al. 2014). Therefore, thermosensitive Ch/Gp hydrogel is a good candidate for implantation of antibiotic loaded polymeric systems directly to the site of infection. The drawback of this injectable chitosan solution is its relatively high initial burst release and high release rate for water soluble drug agents such as vancomycin hydrochloride (Ruel-Gariépy et al. 2000; Gavini et al. 2005). This problem is thought to be minimized by encapsulation of vancomycin in a polymer-based microparticle before inserting in chitosan hydrogel. Microparticles are a favorable technology, as they have been widely used for the sustained and controlled delivery of various drugs (Xiong et al. 2014). Vancomycin has also been incorporated in microparticles, mainly for developing sustained delivery systems. To achieve continuous release and keep an intracameral drug concentration above the minimal inhibitory limit for at least 24 h, vancomycin-loaded-poly(lactide-co-glycolide) microparticles were prepared by Hachicha et al. (2006). In another study, Le Ray et al. (2005) investigated the stability of vancomycin dispersed in poly(ε-caprolactone) microparticles and demonstrated that this combination is a suitable vehicle for the delivery of high local concentrations of vancomycin in an implantation site to avoid systemic side effects. In a recent work, chitosan-based microparticles formulation for colon delivery of vancomycin was prepared using a spray dryer method (Cerchiara et al. 2015). The spray drying process, as an interesting solidification method, received excessive attention for the preparation of drug-loaded microparticles (Gavini et al. 2005). Such success is because of its comparative simplicity, high encapsulation efficiency, and capability to control several parameters such as size and morphology.

In this work, a new composite vancomycin (Van) release system based on hydroxy propyl methyl cellulose (HPMC) microparticles and chitosan thermosensitive hydrogel was fabricated to improve local treatment of osteomyelitis. HPMC is one of the most commonly used hydrophilic biodegradable polymers in the development of controlled release formulations approved by the United States Food and Drug Administration (FDA). Vancomycin-loaded HPMC microparticles (Van-HPMCs) were prepared by spray drying mixtures of vancomycin hydrochloride and HPMC. Then, the Van-HPMCs were embedded in an injectable chitosan/Gp (Ch/Gp) solution to introduce a composite drug release platform (Van/HPMCs-Ch/Gp). The release profile of vancomycin from Van/HPMCs-Ch/Gp was investigated and compared with in vitro release of free vancomycin hydrochloride (Van), vancomycin-loaded Ch/Gp hydrogel (Van-Ch/Gp) and vancomycin-loaded microparticles (Van/HPMCs). The thermosensitivity, hydrogel strength, morphology and degradation rate of Van/HPMCs-Ch/Gp were also investigated.

## Materials and methods

### Materials

Chitosan having an average molecular weight and high degree of deacetylation (DDA = 95%) was purchased from Primex (Iceland). *β*-Glycerolphosphate disodium salt-pentahydrate (Gp, *M*_w_ = 306.12) were obtained from Sigma-Aldrich (St. Louis, USA). Phosphate-buffered saline (PBS, pH = 7.4) was purchased from Gibco (USA). Vancomycin hydrochloride was purchased from Dana Pharmaceutical Company (Iran).

### Preparation of Van-HPMCs

The spray drying method was utilized to prepare HPMC microparticles. The process was performed using a spray drier (Buchi, mini190, Germany), with a standard 0.7 mm nozzle. In the spray drying method, when the solution is injected into the chamber through the nozzle, atomization caused by compressed air breaks down the solution into small droplets. Evaporation of solvent in the droplets occurs as a result of exchanging heat with the blown hot air. The dry product is then collected in a collection bottle. In the standard condition, the inlet temperature, outlet temperature, solution feed rate, aspiration rate and compressed spray air flow were set at 135 °C, 75 °C, 6 ml/min, 75% and 10 L/min, respectively. The Van-HPMCs were prepared by dissolving vancomycin hydrochloride in the HPMC aqueous solution prior to spray drying in three different concentrations, 2.5, 5 and 10 mg/ml.

Size, morphology and surface appearance of the spray-dried HPMC microparticles were studied by scanning electron microscopy (SEM, Philips XL30, Netherlands). The microparticles were placed on a double-sided tape, previously secured on aluminum stubs and then, were observed using SEM at an acceleration voltage of 25 kV after gold sputtering and under argon. The size of the microparticles was measured from the SEM micrographs by ImageJ software (National Institute of Health).

The percentage of production yield (*Y*%) was calculated from the weight of dried microparticles recovered from each of three batches and the sum of the initial dry weight of starting materials:

1 $$ {\text{Production yeid}}\;(Y\% ) = \frac{\text{Weight of dried microparticles}}{{{\text{Total weight of initial drug and polymer}} }}. $$

To evaluate vancomycin content, drug-loaded microparticles were weighed and dissolved in 100 ml of PBS solution (pH = 7.4). The above solution was preserved on high speed stirring for 2 h to get supreme drug released from the particles. Then obtained solution was filtered through Whattman filter paper to eliminate any HPMC residues. The obtained clear solution was analyzed using UV spectrophotometer (Shimadzu, UV1800 Tokyo, JAPAN) at *λ*_max_ value of 280 nm. The drug loading capacity of HPMC microparticles was calculated according to the following equation:

2 $$ {\text{Loading capacity}}\;({\text{LC}}\% ) = \frac{{{\text{The weight of drug in microparticles}} }}{{{\text{Total weight of the microparticles}} }} 100\% . $$

### Preparation of Ch/Gp solutions

The Ch/Gp solution was prepared based on previous studies (Ghasemi-Tahrir et al. 2014). Clear solution of chitosan was prepared by dissolving 200 mg of chitosan in 10 ml aqueous solution of acetic acid (0.1 M) under magnetic stirring for 24 h. The resulting chitosan solution was centrifuged at 10,000 rpm for 30 min to separate the impurities, and the clean chitosan solution was collected. The pH of the acetic acid solution has been adjusted to 4.0 by adding droplets of potassium hydroxide solution (1 M). The chitosan solution was cooled down to 4 °C and continuously stirred while adding 800 mg of Gp powder. Mixing was carried out until a homogeneous Ch/Gp solution was formed. The resulting clear solution containing 2% (w/v) Ch and 8% (w/v) Gp was maintained at 4 °C for further study.

In vitro degradation test of Ch/Gp hydrogel was carried out as follows. About 2 ml of the Ch/Gp solution was added to 25-ml glass tube and heated at 37 °C for 30 min. Next, 10 ml of phosphate-buffered solution (PBS, pH = 7.4) was added to the tube. At predetermined intervals, the hydrogel was removed from the medium, gently blotted and weighed. The weight remaining ratio (WRR %) was calculated using Eq. 3 (Pakzad and Ganji 2016):

3 $$ {\text{WRR}}\% \, = \, \left( {W_{t} /W_{0} } \right) \times 100 $$

where *W*_0_ and *W*_t_ are the first and final weights of the hydrogels, respectively.

### Preparation of Ch/Gp hydrogel containing vancomycin-loaded HPMC microparticles (Van/HPMCs-Ch/Gp)

The Van-HPMCs (16% w/w) were added to the Ch/Gp solution under vigorous stirring. The resultant mixtures were heated at 37 °C until the Van/HPMCs-Ch/Gp hydrogels were formed. In this study, the chitosan hydrogel containing vancomycin hydrochloride (Van-Ch/Gp) was used as control. In this case, free vancomycin hydrochloride (not encapsulated in HPMC microparticles) was added to Ch/Gp solutions under stirring at 4 °C. The resulting solution contained 2% w/v Ch, 8% w/v Gp and 0.1% w/v vancomycin hydrochloride was heated at 37 °C until the Van-Ch/Gp hydrogel was formed.

The morphology and microstructure of the Ch/Gp and Van/HPMCs-Ch/Gp hydrogels were analyzed by SEM (XL30, Philips, Netherlands). Micrographs were recorded at 2.0 mbar H_2_O pressure and 20 kV. For preparation of hydrogels, 1 ml solution of Ch/Gp and Van/HPMCs-Ch/Gp was dumped in a suitable container and became gel at 37 °C and then was immediately frozen at −80 °C. Afterward, the samples were put in a freeze dryer for 3 h at −50 °C and 0.1 mbar and finally, were imaged by SEM.

### Rheological characteristics

Rheological characteristics of the Ch/Gp and Van/HPMCs-Ch/Gp solution were performed using a MCR 300 rheometer (Anton Paar, Austria) equipped with a parallel-plate sensor (25PP) in oscillatory mode. The samples were placed into the rheometer and covered with mineral oil to prevent water evaporation during measurements. Changes in the loss modulus (viscous) and storage modulus (elastic) at 37 °C were determined as a function of time. The measurements were taken by applying a constant frequency and strain values of 1 Hz and 1%, respectively.

### In vitro drug release study

The in vitro release of Van/HPMCs-Ch/Gp hydrogel was studied using the dialysis method. Dialysis bags with molecular weight cut-off (MWCO) of 50 kD were used to retain the Van/HPMCs-Ch/Gp hydrogel and the released Van/HPMCs microparticles, and allow the released vancomycin hydrochloride to permeate into the release medium (PBS, pH 7.4). The Van/HPMCs-Ch/Gp solution was added to the dialysis bag, soaked in PBS at 37 °C and shaken at 90 rpm. At predetermined time intervals, the release medium was sampled and replaced by an equal volume of fresh buffer to maintain a constant volume. The amount of the released vancomycin hydrochloride was determined by UV analysis at 280 nm (Spectrophotometer UV-160A, Shimadzu, Japan). All the measurements were performed triplicate; data are reported as mean ± SD.

The in vitro release of free Van, free Van-Ch/Gp and Van/HPMCs was also studied.

## Results and discussion

### Characterization of vancomycin-loaded HPMC microparticles (Van-HPMCs)

**Fig. 1:**
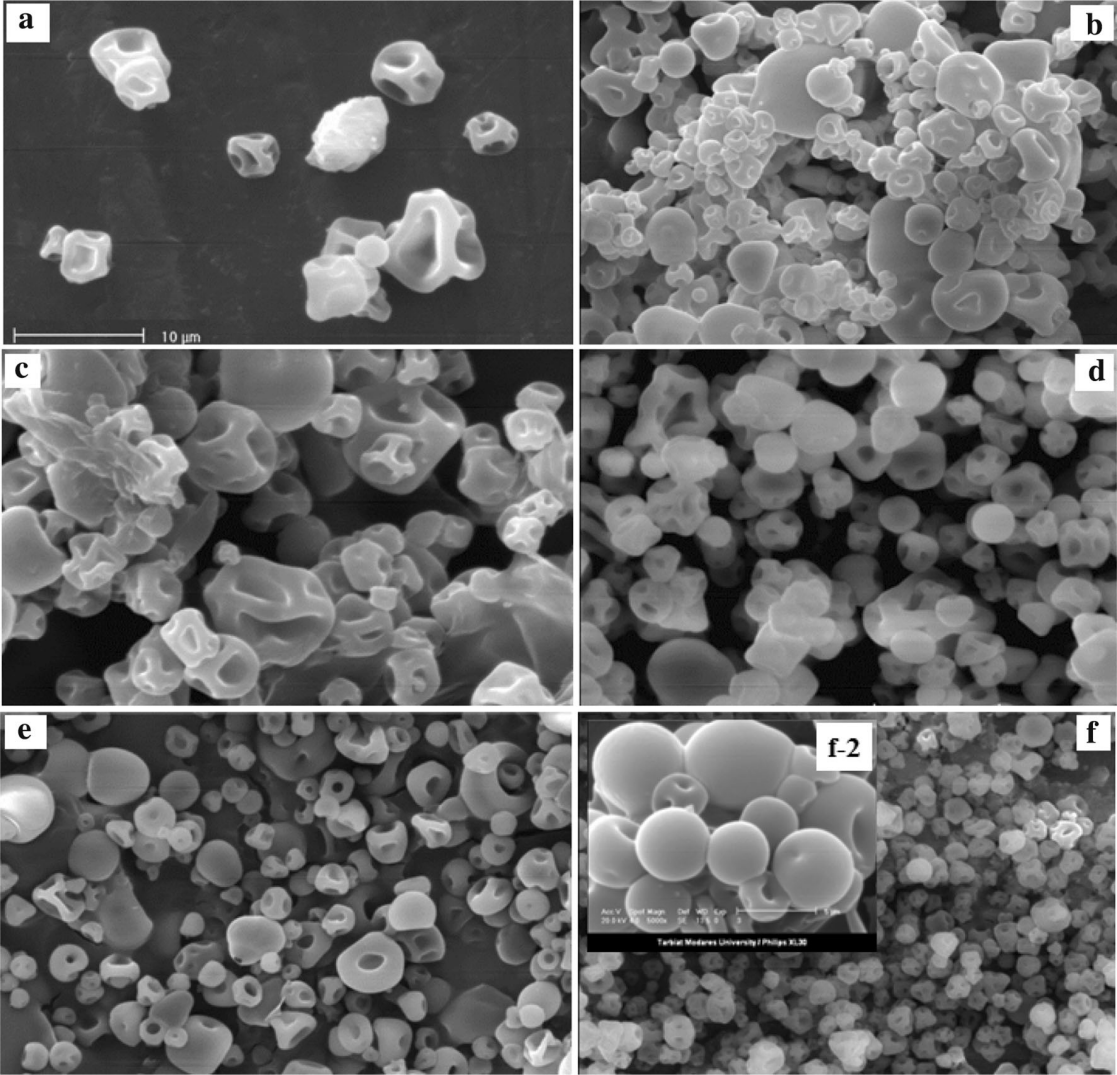
SEM images of spray-dried microparticles, samples H1 (**a**), H2 (**b**), H3 (**c**), H4 (**d**), H5 (**e**) and H6 (**f**) from Table 1, (magnification ×1000, *Scale bar* 10 μm). Selected slice in f-2 is sample H6 with magnification ×5000 (*Scale bar* 5 μm)

The Van-HPMCs with variable polymer concentration and polymer/drug ratio were prepared using the spray drying method (Table 1). The shape and surface morphology of Van-HPMCs were observed using SEM (Fig. 1). As shown in Fig. 1a, the HPMC microparticles without vancomycin hydrochloride with particle diameter range of 3–8 µm (the sample H1 in Table 1) were almost non-spherical, with irregular shape and collapsed structure. Instead, the microparticles formed in the presence of drug (the samples H2 to H4 in Table 1 and Fig. 1) had approximately regular shapes compared to the pure sample. As Fig. 1 shows, by increasing the drug ratio, the surface non-uniformity and the particle size distribution have been decreased (the samples H2 to H4 in Table 1 and Fig. 1b–d). The sample H4 containing 10 mg/ml of HPMC and 10 mg/ml of vancomycin had more spherical structure and lowest particle size distribution. Therefore, the polymer/drug ratio of 1/1 was used for the following studies.

**Table 1:** Characteristics of Van-HPMCs spray-dried microparticles

Sample	Spray dryer feed composition			Obtained microparticles	
	HPMC/drug ratio	HPMC (µg/ml)	Vancomycin (µg/ml)	Particle size (µm)	Surface appearance
H1	–	10	–	3.0–8.0	Completely collapsed
H2	4/1	10	2.5	4.0–22	Spherical—collapsed
H3	2/1	10	5.0	3.0–18	Spherical—rough
H4	1/1	10	10	2.0–13	Spherical—smooth
H5	1/1	5.0	5.0	2.5–10	Smooth
H6	1/1	0.5	0.5	1.5–6.4	Spherical and uniform

The effect of HPMC solution concentration on the microparticles properties was also studied by using three different HPMC concentrations (10, 5 and 0.5 mg/ml for the samples H4, H5 and H6, respectively). It is evident that with decreasing HPMC concentration, the particle size and surface non-uniformity decreased (Table 1; Fig. 1d–f). The effect of HPMC solution concentration on production percent yield (Y%) and drug loading capacity (LC) of the Van-HPMCs was also of inverse type (Table 2). With decrease in the polymer solution concentration, the percent yield and drug loading increase. Finally, the sample H6 (0.5 mg/ml HPMC concentration and polymer drug ratio of 1/1) with completely spherical and smooth structure, lowest particle size (1.5–6.4 μm) and highest loading capacity (63%) and production yield (33%) was used as the best Van-HMPCs in the next studies.

**Table 2:** Drug loading capacity and production yield of Van-HPMCs spray-dried microparticles

Sample	Drug loading capacity (LC%)	Production yield (*Y*%)
H4	51.70	12
H5	55.74	21
H6	62.87	33

### Characterization of Ch/Gp hydrogels containing vancomycin-loaded HPMC microparticles (Van/HPMCs-Ch/Gp)

**Fig. 2:**
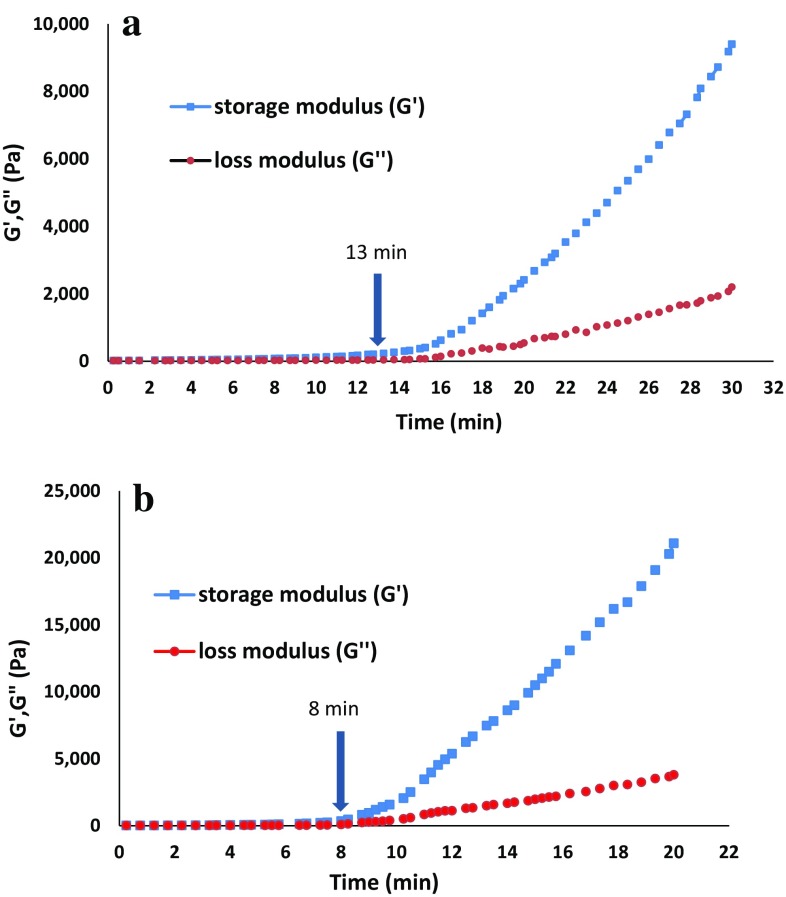
Time dependence of storage modulus (*G*′) and loss modulus (*G*″) of **a** Ch/Gp solution (2% chitosan & 8% Gp) and **b** Van/HPMCs-Ch/Gp) solution (2% chitosan, 8% Gp & 16% Van/Hpmcs at 37 °C, the gelling times for this hydrogels were **a** 13 min and **b** 8 min

Chitosan solution containing glycerolphosphate disodium salt (Gp) is an injectable thermosensitive in situ gel-forming system which undergoes sol–gel transition under physiological pH and temperature. Based on our previous studies, the gelation time of this solution is completely influenced by molecular weight, deacetylation degree and concentration of chitosan, and Gp salt concentration (Ghasemi-Tahrir et al. 2014; Khodaverdi et al. 2013; Ganji et al. 2007). Therefore, Ch/Gp solution containing 2% (w/v) of chitosan with an average molecular weight and high degree of deacetylation (DDA = 95%), and 8% (w/v) of Gp was used in this study. This choice showed adequate thermosensitivity, good injectability and storage stability. Since the Ch/Gp solution is basically designed to be used via parenteral injection, it is necessary to have precise determination of its gelation time in the presence of HPMC microparticles. Gelling time of the Ch/Gp and Van/HPMCs-Ch/Gp was evaluated by measuring the storage modulus (*G*′) and loss modulus (*G*″) at 37 °C versus time. Figure 2a shows the rheological behavior of the Ch/Gp sample during sol-to-gel transition at 37 °C. At first, *G*′ and *G*″ presented nearly constant values showing that viscous behavior was dominant before gelation occurred. As time passed, the values for *G*′ increased to values greater than those for *G*″; however, these two moduli reached equal values at the gelation point. After the gelation point, *G*′ increased to a much higher value, as compared to *G*″. It exhibited a solid-like behavior in which elastic behavior was dominant. The same behavior was recorded for Van/HPMCs-Ch/Gp in Fig. 2b. Evidently, after embedding of HPMC microparticles in the Ch/Gp hydrogel, the gelation time sufficiently decreased from 13 to 8 min.

**Fig. 3:**
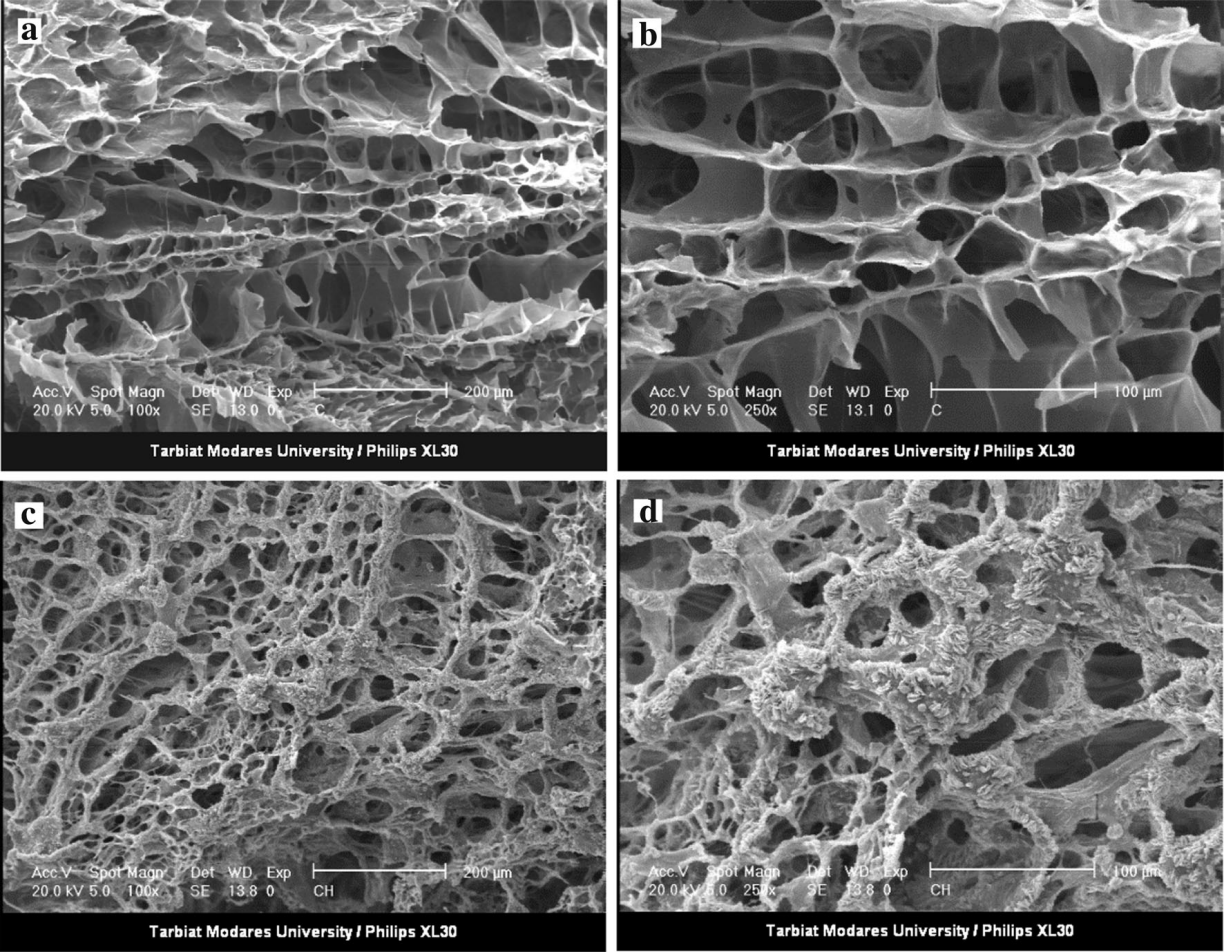
SEM images of Ch/Gp hydrogel (**a** magnification ×100, **b** magnification ×250), and Van/HPMCs-Ch/Gp hydrogel (**c** magnification ×100, **d** magnification ×250)

Figure 3a, b shows the SEM results for the morphology of these hydrogels. As can be seen, the Ch/Gp hydrogel has a spongy and porous structure with a smooth matrix surface. Interestingly, this permeable structure in the Van/HPMCs-Ch/Gp hydrogel was also observed (Fig. 3c, d). Instead, a rough and unsmooth surface is observed in the Van/HPMCs-Ch/Gp hydrogel which could be attributed to the presence of Van/HPMCs in the hydrogel structure.

### In vitro drug release

**Fig. 4:**
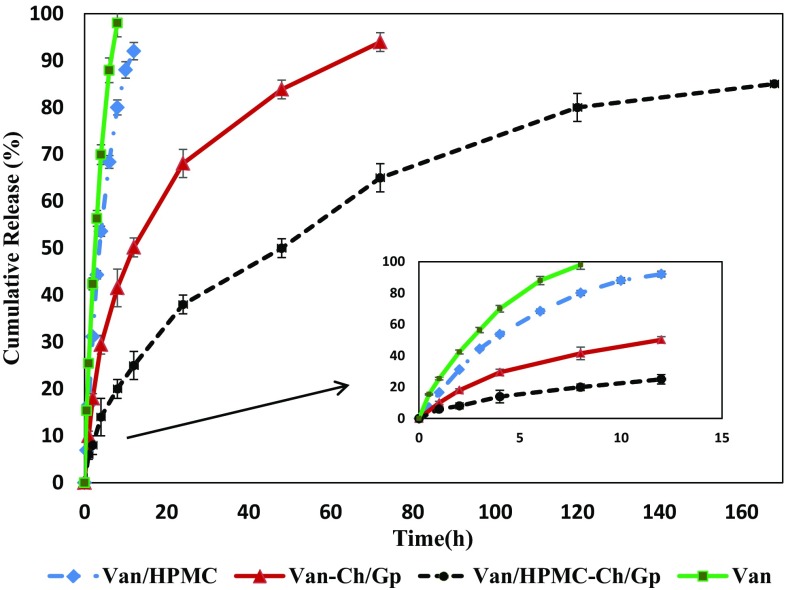
Cumulative vancomycin hydrochloride release profiles in PBS (*n* = 3)

The release profiles of free Van, Van-Ch/Gp, Van/HPMCs and Van/HPMCs-Ch/Gp are shown in Fig. 4. The release of free Van was very fast (99% of total drug was released within 8 h). Considering low molecular weight of vancomycin hydrochloride, these results show that the dialysis bag had no retaining effect on drug release. Almost same results were obtained for Van/HPMCs, where 80% of total drug was released within 8 h, and 92% was released within 12 h. This high initial burst and fast release rate could be attributed to the high hydrophilicity of HPMC microparticles. The release rate of Van-Ch/Gp was, however, meaningfully reduced (42% of total drug was released within 8 h, and 94% was released within 72 h), suggesting that the Ch/Gp hydrogel had a noticeable effect on extending vancomycin release. Generally, the drug release behavior from polymeric hydrogels can be attributed to three stages: (1) dissolution of the absorbed drug on the hydrogel surface (the main reason for the burst release), (2) drug penetration through the hydrogel matrix and diffusion within the pores of the media, and (3) thorough degradation of hydrogel and release of the remaining drug.

Considering the above platform, the relatively high initial burst release from Van-Ch/Gp indicated that some of the drug was adsorbed onto the surface or distributed among the pores of the hydrogel during gelation and then, diffused rapidly when the hydrogel came into contact with the release medium (Stage 1). After the initial burst release, the vancomycin trapped in the Ch/Gp hydrogel was released slowly, essentially due to the obstruction effect of Ch/GP hydrogel matrix. The release rates decreased as time passed, which could be attributed to drug decline in the hydrogel matrix and a decrease in the concentration gradient of the drug (Stage 2).

**Fig. 5:**
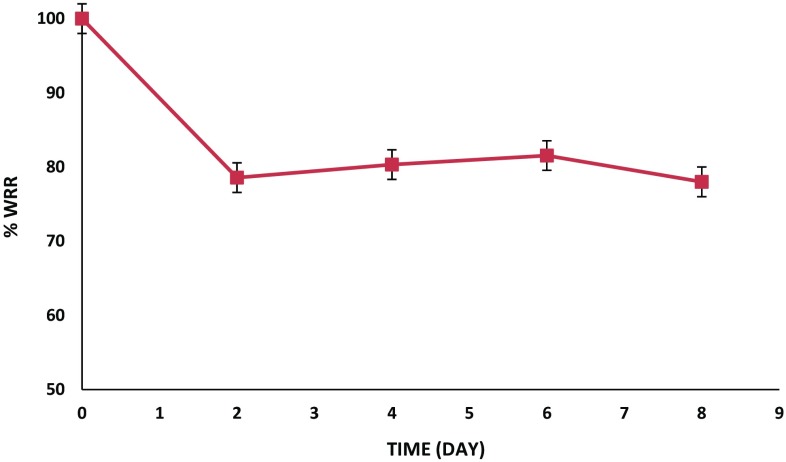
The percentages of wet weight remaining ratio of the Ch/Gp hydrogel incubated in PBS (pH 7.4) at 37 °C as a function of time. Each point represents mean ± SD (*n* = 3)

**Fig. 6:**
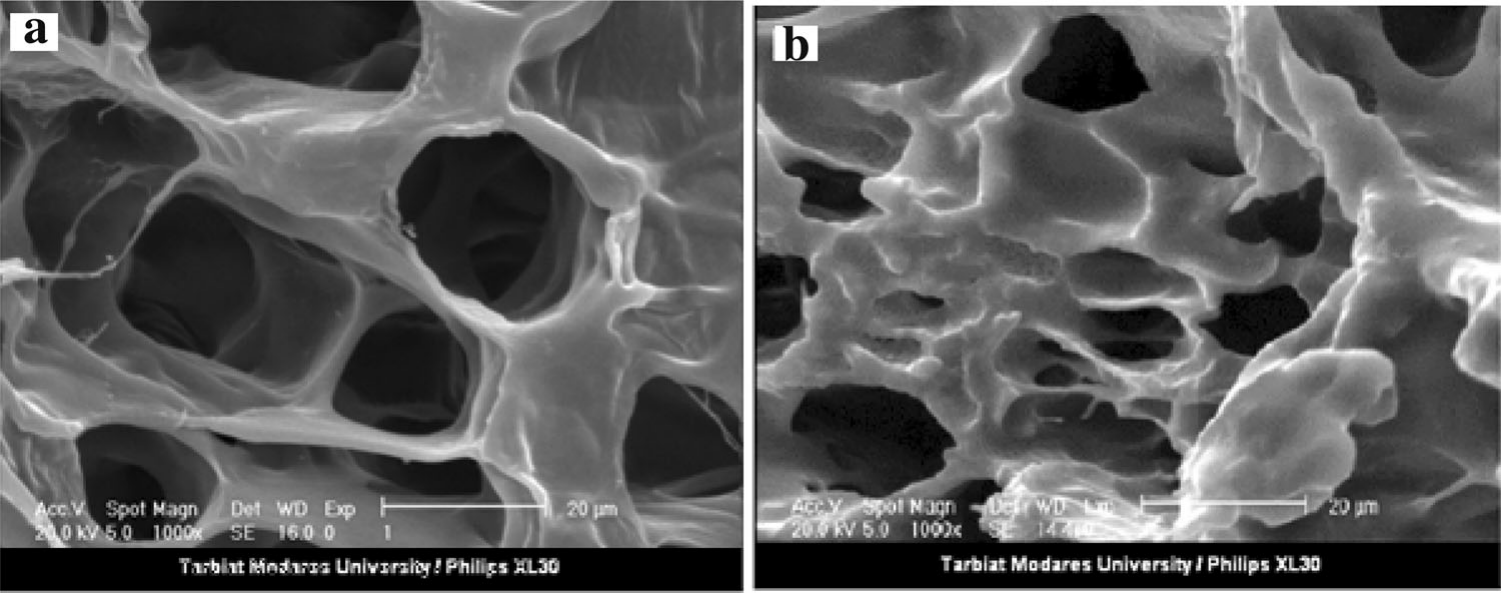
SEM image of Ch/Gp hydrogel **a** before immersion in PBS (magnification ×1000) and **b** after immersion in PBS (pH 7.4 for 8 days, magnification ×1000)

To investigate the possible effect of the hydrogel degradation phenomenon on drug release behavior, the hydrogel weight loss was studied in PBS at 37 °C (Fig. 5). As seen, 20% of the weight of the Ch/Gp was lost in the first 48 h. The significant initial rate of decrease in hydrogel weight occurred as the result of leaching and dissolution of the free Gp salt accumulated in the surface layers of the hydrogels. The weight remaining ratio (WRR %) remained constant during next 8 days. The SEM analysis showed that the inner structure of the Ch/Gp hydrogel did not considerably change during this period, and its inner spongy structure still existed (Fig. 6). Therefore, it can be concluded that drug release from the Ch/Gp hydrogel is only controlled by a diffusional mechanism.

Interestingly, the release rate of Van/HPMCs-Ch/Gp further decreased (20% of total vancomycin was released within 8 h, 25% was released within 12 h, and 85% was released within 160 h), implying that embedding vancomycin-loaded microparticles in chitosan hydrogel played an imperative role in delaying drug release (Fig. 4). In this case, the drug needs to overcome first the barrier of microparticles (diffusion through HPMC microparticles) and then, the barrier of hydrogel matrix (diffusion through chitosan hydrogel). Comparing release profile of Van-Ch/Gp and Van/HPMCs-Ch/Gp in Fig. 4 indicates that the release-slowing down influence of HPMC microparticles is much more imperative than that of the Ch/GP hydrogel. Theoretically, the release rates would be decreased through a composite drug release platform, which consists of microparticles containing drugs embedded within hydrogel matrix. Such a system can be expected to be used in other forms of antibiotic delivery.

## Conclusion

In this work, a new composite vancomycin hydrochloride release system based on HPMC microparticles and chitosan hydrogel was designed for the aim of local treatment of osteomyelitis. The injectable chitosan/Gp thermosensitive solution containing vancomycin-loaded HPMC microparticles showed a rapid transition from solution to gel at 37 °C. Interestingly, this hydrogel, with porous and spongy structure, has a long-term release profile in vitro. Therefore, the biocompatible and injectable Van/HPMCs-Ch/Gp hydrogels have great potential for use in extra sustained antibiotic delivery, by which the injection frequency could be expressively reduced.
